# Prediction of gastrointestinal toxicity after external beam radiotherapy for localized prostate cancer

**DOI:** 10.1186/s13014-015-0389-5

**Published:** 2015-04-08

**Authors:** Vittoria D’Avino, Giuseppe Palma, Raffaele Liuzzi, Manuel Conson, Francesca Doria, Marco Salvatore, Roberto Pacelli, Laura Cella

**Affiliations:** Institute of Biostructure and Bioimaging, National Council of Research (CNR), Naples, Italy; Department of Advanced Biomedical Sciences, Federico II University School of Medicine, Naples, Italy

**Keywords:** NTCP, Radiation induced rectum toxicity, LKB model, Multivariate model

## Abstract

**Background:**

Gastrointestinal (GI) toxicity is a common effect following radiation therapy (RT) for prostate cancer. Purpose of the present work is to compare two Normal Tissue Complication Probability (NTCP) modelling approaches for prediction of late radio-induced GI toxicity after prostate external beam radiotherapy.

**Methods:**

The study includes 84 prostate cancer patients evaluated for late rectal toxicity after 3D conformal radiotherapy. Median age was 72 years (range 53-85). All patients received a total dose of 76 Gy to the prostate gland with daily fractions of 2 Gy. The acute and late radio-induced GI complications were classified according to the RTOG/EORTC scoring system. Rectum dose-volume histograms were extracted for Lyman-Kutcher-Burman (LKB) NTCP model fitting using Maximum Likelihood Estimation. The bootstrap method was employed to test the fit robustness. The area under the receiver operating characteristic curve (AUC) was used to evaluate the predictive power of the LKB and to compare it with a multivariate logistic NTCP model previously determined.

**Results:**

At a median follow-up of 36 months, 42% (35/84) of patients experienced grade 1-2 (G1-2) acute GI events while 25% (21/84) of patients developed G1-2 late GI events. The best-estimate of fitting parameters for LKB NTCP model for mild\moderate GI toxicity resulted to be: *D*_*50*_ = 87.3 Gy, *m* = 0.37 and *n* = 0.10. Bootstrap result showed that the parameter fit was robust. The AUC values for the LKB and for the multivariate logistic models were 0.60 and 0.75, respectively.

**Conclusions:**

We derived the parameters of the LKB model for mild\moderate GI toxicity prediction and we compared its performance with that of a data-driven multivariate model. Compared to LKB, the multivariate model confirmed a higher predictive power as showed by the AUC values.

## Background

To predict the normal tissue complication probability after a radiotherapy (RT) treatment one of the most well-known and used method is the Lyman-Kutcher-Burman (LKB) model [1] based on dosimetric data available from dose-volume histograms.

Several authors have published studies about fitting of LKB model for radiation induced rectal toxicity and many of them considered rectal bleeding (G1-2) as toxicity endpoint. Burman et al. were the first authors who computed the model parameters suggesting the values *D*_*50*_ = 80.0 Gy, *m* = 0.15, *n* = 0.12 for late rectal bleeding [2]. More recently, Rancati et al. [3] have found *D*_*50*_ = 81.9 Gy, *m* = 0.19, *n* = 0.23 and QUANTEC recommended the values *D*_*50*_ = 76.9 Gy, *m* = 0.13, *n* = 0.09 to apply LKB model for the same endpoint [4-6]. While many studies have been published about NTCP models for late rectal bleeding, so far very few authors have reported results about moderate/mild toxicity as high stool frequency, loose stools and rectal urgency as endpoints [7-10].

The traditional NTCP models use only the dose information to predict toxicity. In the last few years several studies have been published concerning the probability of rectal injury depending on many individual factors extracted from clinical information: the drug prescription (anti-hypertensives and/or anti-coagulants), smoking history, previous abdominal surgery, pre-treatment morbidities (hypertension, cardiovascular history), diabetes mellitus, presence of acute gastro-intestinal toxicity [8,11-15]. Defraene et al. demonstrated the benefit of including clinical factors in the predictive power of different NTCP models for all the endpoints considered, i.e. rectal bleeding, high stool frequency and fecal incontinence [8].

In a previous work, we derived a multivariate NTCP model for late GI toxicity from a set of 57 patients treated with RT for localized prostate cancer [15] and we compared its performance with the LKB model using the parameters suggested in literature [7]. In the present work we extended the sample size with an additional dataset up to 84 patients in order to: 1) fit the LKB model deriving the parameters for specific rectal toxicity; 2) validate the multivariate model on an extended cohort of patients and compare its predictive power with the LKB model.

## Methods

### Clinical and dosimetric data

We retrospectively analyzed the treatment plans of 99 consecutive patients affected by localized prostate adenocarcinoma treated with radiotherapy at the radiation oncology department of the University “Federico II” of Naples. Data of radiation-induced rectal toxicity and treatment characteristics were obtained from clinical reports and matched with the dosimetric data. All participants gave written informed consent and the patient data were analyzed anonymously. This retrospective study was approved by the local Ethics Committee. For 14 out of 99 patients physics and/or follow-up data were not available while one patient experienced GI disorders before treatment. These patients were excluded from further evaluation. Clinical data included cardiac comorbidities, smoking history, hormonal therapy, drugs prescription, prostate specific antigen (PSA) at diagnosis. Dosimetric data were extracted from the cumulative dose-volume histograms (DVHs).

All patients were treated with full three-dimensional radiation treatment planning with a total dose of 76 Gy in 38 daily fractions of 2 Gy. RT was administered with 20 MV photon beams from linear accelerator by conformal radiation technique (CRT) with six field arrangement or by conformal dynamic arc radiation technique (ART). XIO (Elekta CMS) and ERGO (3D-Line Medical System) treatment planning systems were used. The above RT techniques are described more in detail in [15].

Treatment planning was based on computed tomography (CT) performed with empty rectum, comfortably filled bladder, and with the patient in prone position using vacuum-locked mattress. Five-millimeter increment CT slices of the pelvis extending from L4-L5 to 2 cm caudal to the bottom of ischial tuberosities were acquired. CT images were electronically transferred to the CT simulation software (Focal Ease 4.2, Elekta CMS) for target and critical organs contouring. Clinical target volume (CTV) included the prostate gland or the prostate gland plus the seminal vesicles. A 1 cm margin was 3D automatically added around the CTV to define the planning target volume (PTV), except at the boundary between the anterior rectal wall and the prostate, where a 0.5 cm margin was used. The rectum delineation was performed on purpose by the same radiation oncologist (M.C.) according to the male Radiation Therapy Oncology Group (RTOG) Normal Pelvis Atlas [16]. The prescription dose was specified at the center of the PTV. Field weightings were adjusted to achieve the 95% of prescription dose to 95% of the PTV.

### End point

The definition of acute and late GI toxicity was evaluated according to Radiation Therapy Oncology Group/European Organization for Research and Treatment of Cancer (RTOG/EORTC) criteria [17]. GI treatment toxicity was classified as acute if it occurred during radiotherapy and in the first 3 months thereafter, while it was classified as late GI toxicity if present after 3 months from the completion of treatment. The monitoring of patients for GI toxicity was part of clinical routine, follow-up visits were planned every 3 months for the first year, then every 6 months for the next 3 years, and yearly thereafter.

### Statistical analysis and modelling

Univariate logistic analysis was performed using the Spearman’s rank correlation (R_s_) coefficient to assess correlation of patient clinical data with late GI toxicity.

#### Normal tissue complication probability models

In this study, we have analyzed two NTCP models: Lyman-Kutcher-Burman (LKB) model and multivariate logistic NTCP model.

According to LKB model with generalized equivalent uniform dose (gEUD) formulation, NTCP is expressed by the following equations [18]:

1 $$ NTCP=\frac{1}{\sqrt{2\pi }}\kern0.5em {\displaystyle \underset{-\infty }{\overset{t}{\int }}{e}^{-\frac{x^2}{2}}}dx $$

2 $$ t=\frac{gEUD-{D}_{50}}{m\kern0.5em {D}_{50}} $$

3 $$ gEUD={\left({\displaystyle \sum_i{v}_i\kern0.5em {d_i}^{\frac{1}{n}}}\right)}^n $$

*D*_*50*_ is the value of the dose corresponding to the 50% probability to induce normal tissue complication, the parameter *m* is inversely proportional to the slope of dose-response curve, the parameter *n* can assume values in the range 0-1 and accounts for volume effect of the organ; *v*_*i*_ is the relative volume that receives the dose *d*_*i*_, the sum is over all the bins of DVH.

The Maximum Likelihood (ML) method was used to find the best-fit values of the parameters *D*_*50*_, *m* and *n* by maximizing the logarithm of the likelihood (LLH):

4 $$ LLH\kern0.5em \left({D}_{50},\kern0.24em m,\kern0.24em n\right)={\displaystyle \sum_{y(i)=1} \log \kern0.5em \left( NTCP\kern0.5em \left({D}_{50},\kern0.24em m,\kern0.24em n\right)\right)\kern0.5em +}\kern0.5em {\displaystyle \sum_{y(i)=0} \log \kern0.5em \left(1- NTCP\kern0.5em \left({D}_{50},\kern0.24em m,\;n\right)\right)} $$

The sum is over all the patients with different outcome y(i) = 1 and y(i) = 0, i.e. with and without GI toxicity respectively.

The LLH function was numerically maximized by the Nelder-Mead Simplex Method (Matlab implementation: FMINSEARCH function) using an in-house developed library for Matlab [19].

Ninety-five percent confidence intervals for parameters estimates were obtained using the profile likelihood method [20]. Following this method, each parameter belonging to the set (*D*_*50*_, *m, n*) was varied around its ML estimate (optimum LLH) while the other two parameters were fixed at their ML estimate.

The 95% confidence bounds were determined reducing the maximum LLH by one half of the χ^2^ inverse cumulative distribution function associated to 95%, so as to obtain the iso-likelihood contours in each Cartesian plane of the parameters space (*D*_*50*_*, m, n*). In correspondence to the parameters values belonging to the iso-likelihood contours, a bundle of NTCP curves was calculated and the 95% confidence region for the model fit was thus estimated [21].

In order to perform an internal validation of the fitting results and to test the fit robustness, the bootstrap method was here employed to determine the spread in ML estimation of NTCP parameters. The bootstrap resampling method works by refitting the NTCP model using the ML estimation to many pseudo-datasets which are created by subsampling the input data set (20000 bootstrap resample runs with a number of folds of 80).

The logistic regression model is based on the sigmoidal relationship between dose and response endpoint. The normal tissue complication probability is given by:

5 $$ NTCP=\frac{e^{g(x)}}{1\kern0.5em +\kern0.5em {e}^{g(x)}} $$

where *x* represents a vector of input variables and *g(x)* is given by the following equation:

6 $$ g(x)=\alpha \kern0.5em +{\displaystyle \sum_{i=1}^s{\beta}_i}\kern0.5em {x}_i\;i=1,\dots, s $$

The model order is defined as *s*, while α and β_i_ are the corresponding set of model coefficients determined by maximizing the probability that the data gave rise to the observed outcomes.

#### Model’s evaluation and comparison

In a previous analysis of the GI toxicity on a subset (57 patients) of the present dataset we developed a 3-variable logistic regression model consisting of the percentage of rectum volume which receives at least a dose of 65 Gy (V65), the use of antihypertensive and/or anticoagulant drugs (AH/AC) and the manifestation of acute GI toxicity. The expression of *g(x)* with the values of the coefficients is given by:

7 $$ g(x)=\kern0.5em -1.283\kern0.5em +\kern0.5em 0.028\kern0.5em \times \kern0.5em V65\kern0.5em -\kern0.5em 1.442\kern0.5em \times \kern0.5em AH/ AC\kern0.5em +\kern0.5em 1.458\kern0.5em \times \kern0.5em  Acute\kern0.5em GI\kern0.5em  toxicity $$

To assess the correlation of the LKB and the above multivariate model with GI toxicity the R_s_ coefficient was analyzed. To evaluate the discriminating ability of model fits, the receiver-operating characteristic (ROC) analysis was performed and the area under the receiver operating characteristic curve (AUC) was calculated (SPPS Inc., Chicago IL, vs. 18). The discrimination value on the ROC curve, i.e. the cut-off point optimally classifying patients in a binary prediction problem, was determined by Youden’s J statistic [22]. ROC curve comparison was performed by a Z test.

## Results

Clinical and dosimetric data were collected for eighty-four patients of which 42% (35/84) had developed acute GI toxicity while 25% (21/84) had developed late GI toxicity. Among cases of acute rectal morbidity, 74% (26/35) were G1 and 26% (9/35) were G2. Of note, no cases of late rectal toxicity greater than grade 1 were reported.

The univariate logistic regression analysis showed a negative relation between antihypertensive/anticoagulant treatments and GI late toxicity, while a positive relation was found between acute GI toxicity and late GI toxicity (Table 1).

**Table 1:** **Main clinical data, summary of univariate logistic regression and correlation coefficient (R**_**s**_ **) with radiation-induced late gastro-intestinal toxicity incidence**

				**Univariate analysis**	
**Clinical characteristic**		**N**	**%**	**R**_**s**_	**p-value**
Age (yrs)					
≤70		36	42.9		
>70		48	57.1	0.167	0.130
Antihypertensive/anticoagulants					
Yes		56	66.7		
No		28	33.3	-0.292	0.007
Antihypertensive					
Yes		53	63.1		
No		31	36.9	-0.242	0.026
Anticoagulants					
Yes		34	40.5		
No		50	59.5	-0.140	0.204
Acute GI toxicity					
Yes		35	41.7		
No		49	58.3	0.293	0.007
	Grade				
	0	49	58.3		
	1	26	31.0		
	2	9	10.7		

**Figure 1:**
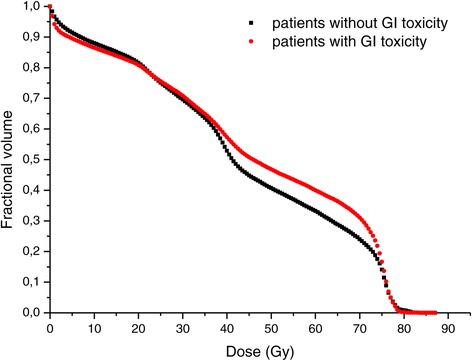
**Average cumulative rectum DVH for patients who have developed GI toxicity (red line) and for patients who haven’t developed GI toxicity (black line).**

With regard to the dosimetric evaluation, in Figure 1 the mean cumulative rectum DVHs for GI late toxicity patients and complication-free patients were compared: on average, the rectal volume irradiated in the dose range of 25-70 Gy for GI late toxicity patients was greater.

### LKB fitting

**Figure 2:**
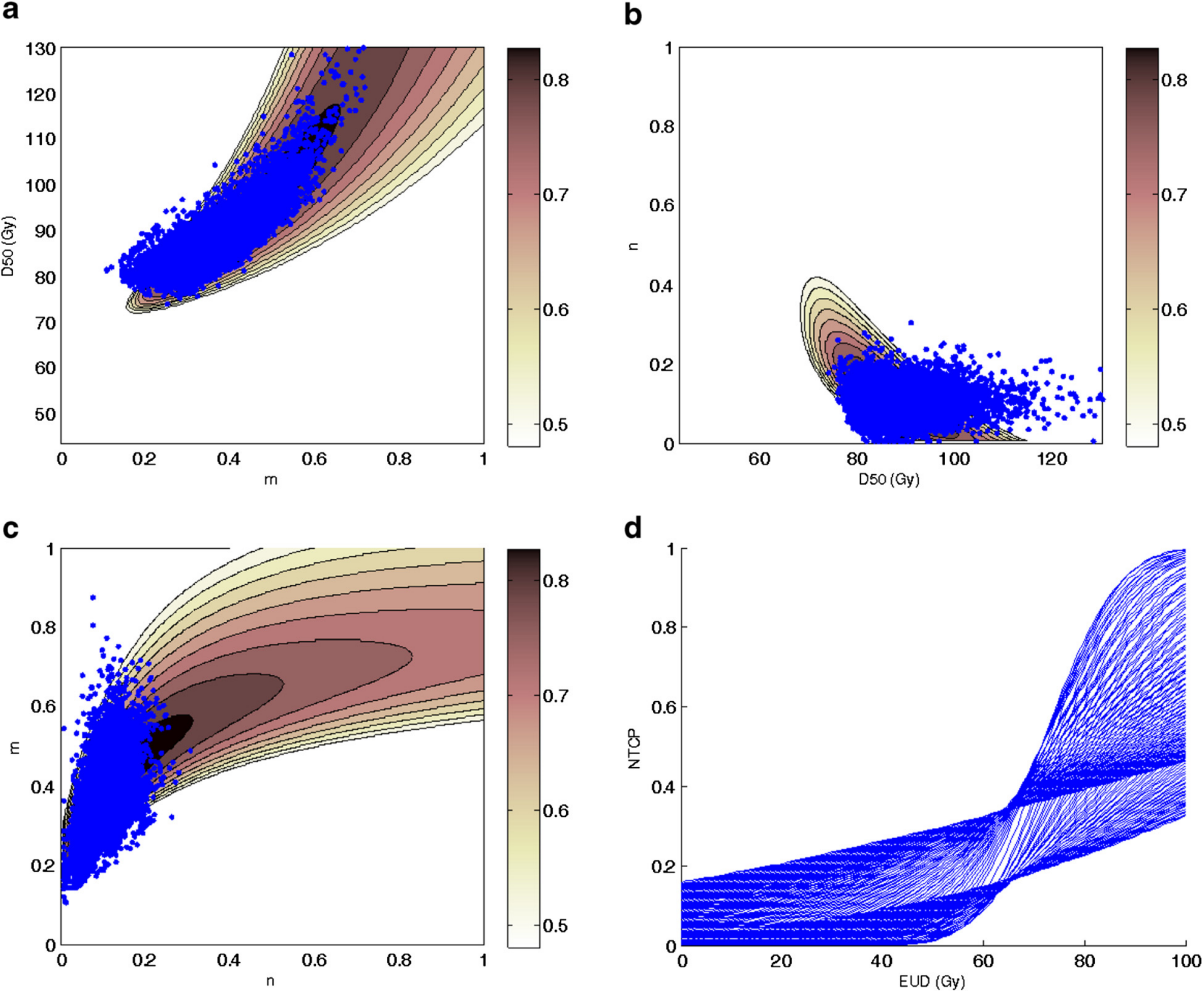
**Likelihood estimation values plotted as a function of rectum LKB parameters: a)** ***m*** **and** ***D***_***50***_ **for fixed value of** ***n*** **= 0.10; b)** ***D***_***50***_ **and** ***n*** **for a fixed value of** ***m*** **= 0.37; c)** ***n*** **and** ***m*** **for a fixed value of** ***D***_***50***_ **= 87.3 Gy; d) NTCP bundle curves showing 95% confidence interval region fit for the model. Blue points represent the results of bootstrap resample runs.**

The optimal NTCP parameters values for LKB model resulted to be *D*_*50*_ = 87.3 Gy (95% CI 75.9-102.2 Gy), *m* = 0.37 (95% CI 0.26-0.64), *n* = 0.10 (95% CI 0.02-0.26) and the corresponding value of log-likelihood is LLH = -46.3. Figures 2a-c illustrate the iso-likelihood contours in each Cartesian plane of the parameters space (*D*_*50*_, *m, n*). In Figure 2d the bundle of NTCP curves corresponding to the 95% confidence interval region for the model fit is plotted.

**Figure 3:**
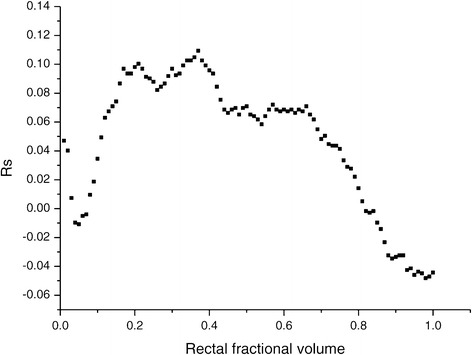
**Plot of correlation coefficient of LKB NTCP values as a function of the volume fraction of rectum corresponding to the D**_**x**_ **dose.**

The optimal parameters were used to calculate the NTCP values as a function of D_x_ doses, being D_x_ the minimum dose to the x volume. The NTCP values and the fractional rectal volume were then correlated using the R_s_ coefficients (Figure 3).

To test the fit robustness we performed a bootstrap method. The mean and the standard deviation of LKB NTCP model parameters obtained for bootstrap samples are *D*_*50*_ = 87 Gy (SD = 6 Gy), *m* = 0.37 (SD = 0.08), *n* = 0.10 (SD = 0.03). The mean values of *m* and *n* parameters are close to the exact fit to the whole patient cohort.

### Comparison of the predictive capability of LKB and logistic NTCP models

In a previous work we suggested a three-variable logistic NTCP model, including clinical patient-specific factors [15]. Accordingly, the risk of G1-2 late GI toxicity increased as V65 increased, it was higher for patients experiencing previous acute toxicity and lower for patients taking antihypertensive and/or anticoagulant drugs. The model exhibited a good predictive performance (AUC = 0.79). When applied to the present extended dataset, the logistic NTCP performance is still good with an AUC value of 0.75 (95% CI 0.613-0.891).

**Figure 4:**
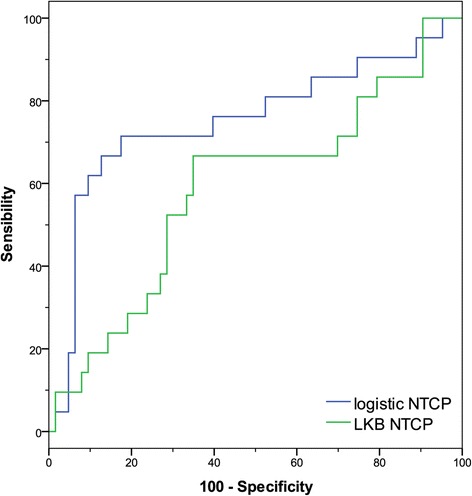
**ROC comparison. Logistic regression model vs. LKB model for gastrointestinal toxicity.**

For comparison, the AUC and R_s_ values of the multivariate logistic and LKB models are reported in Table 2 and the ROC curves are shown in Figure 4.

**Table 2:** **AUC values of ROC curves and Spearman’s correlation coefficient (R**_**s**_ **) of LKB and logistic NTCP models with 95% confidence intervals**

**Model**	***AUC***	***R***_***s***_
**LKB**	0.60 (0.442, 0.736)	0.133
**Logistic**	0.75 (0.613, 0.891)	0.378

**Figure 5:**
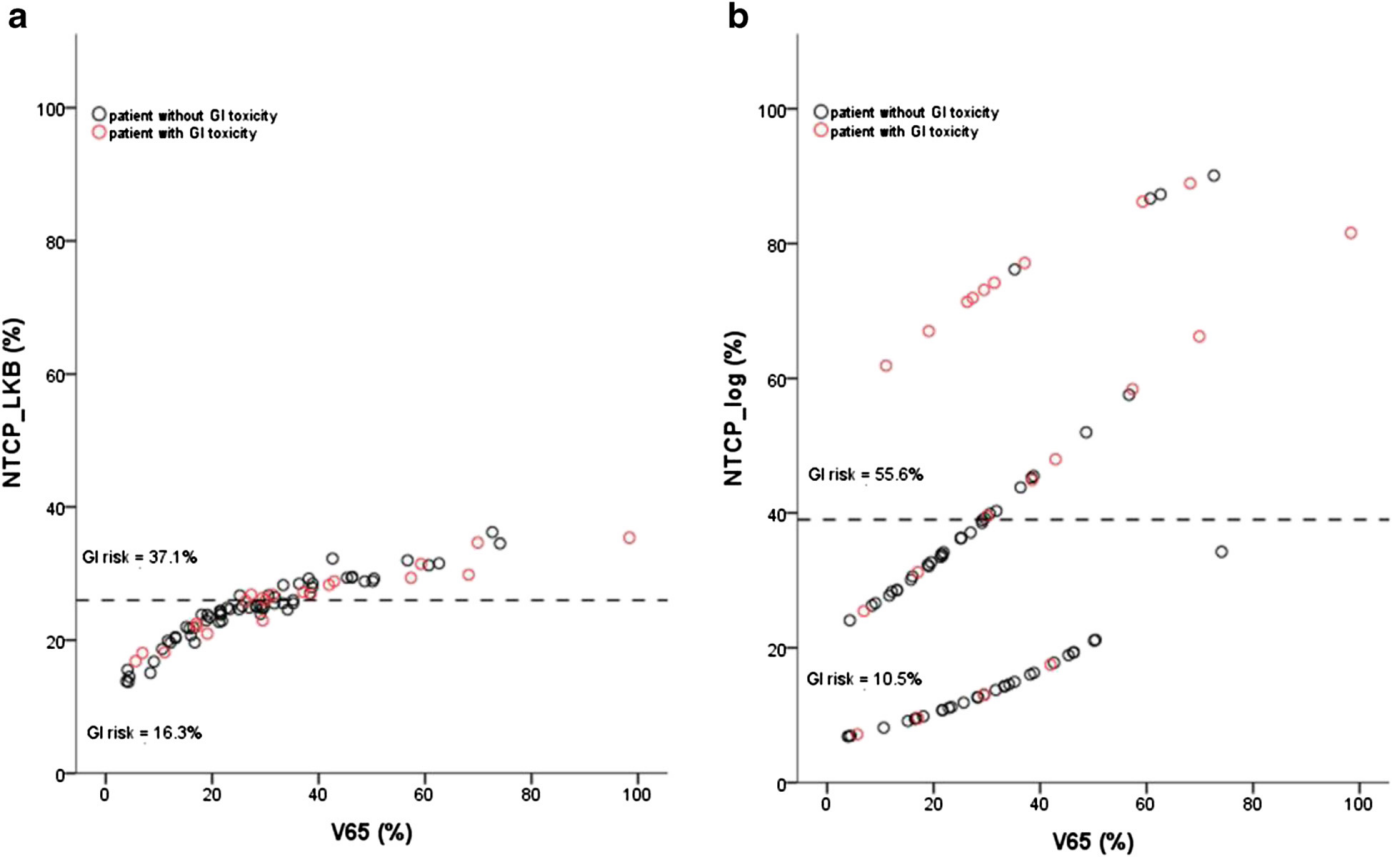
**Scatterplot of V65 (%) for the rectum vs. normal tissue complication probability (NTCP, %) for LKB and logistic models ((a) and b) respectively).** Patients with gastrointestinal (GI) toxicity are plotted as open red circles, the patients free from GI toxicity are represented as open black circles. For the LKB model the region with NTCP < 26% had a lower incidence of GI toxicity than the rest of the region, while for logistic model the region with a lower incidence of GI toxicity is that down NTCP value of 39%.

The discrimination value on the ROC curve for LKB-NTCP model is 26% and for the logistic NTCP model is 39%. According to LKB model, GI toxicity incidence was higher in patients with NTCP ≥ 26% than in those with NTCP < 26% (37.1% vs. 16.3%) (Figures 5.a and b). According to the logistic model GI toxicity occurred more frequently in the group with NTCP ≥ 39% than in those with NTCP < 39% (15/27, 55.6% vs. 6/57, 10.5%).

The ROC curve comparison showed a significant difference in the prediction capability of the two models: the logistic NTCP resulted in being significantly more predictive when compared to LKB NTCP (z > 1.96, P < 0.05).

## Discussion

In recent years, technological advances in radiation therapy have allowed to deliver higher prescribed dose for localized prostate cancer [23] and to reduce the risk of severe adverse effects. Consequently, we are observing a change in the toxicity profiles of external beam RT and the focus of toxicity analysis is changing from rectal bleeding to quality of life assessment [24]. Alterations of intestinal motility and peristalsis such as high stool frequency, loose stools and rectal urgency can greatly affect the quality of life of long-surviving patients. As a consequence it is extremely important to model different and specific aspects of rectal toxicity in a robust way.

Many studies report on LKB normal tissue complication probability parameters for rectal bleeding [25,26] while very few data are available on late mild/moderate radio-induced toxicity for the rectum. Gulliford et al. [7] have found the LKB parameters for specific rectal complications observed in clinical practice including stool frequency, loose stools and rectal urgency.

In the present study, we retrospectively reviewed 84 patients undergoing radiotherapy for prostate cancer. The most frequently observed symptoms were high stool frequency, loose stools and rectal urgency while no rectal bleeding was recorded.

Using the mild/moderate rectal late toxicity as endpoint, two different modelling approaches were compared: the Lyman-Kutcher-Burman (LKB) and the data-driven multivariate logistic NTCP models. We compared the predictive power of the two models to understand the benefits of a data-driven approach to NTCP modeling.

Indeed in a previous study [15], we have analyzed dosimetric and clinical data of a subset of patients (57) with localized prostate cancer treated with radiation therapy and we have obtained a multivariate normal tissue complication model for the rectum. We compared its predictive performance with the LKB model using a set of parameters proposed in the literature [7]. In order to be fair in comparing two different model philosophies, in the present study based on an extended cohort, we first identified the best set of parameters for the LKB model on our dataset and then we compared its performance with the logistic NTCP model. Figure 4 shows the better performance of multivariate NTCP model (R_s_ = 0.378, p < .001, AUC = 0.751) compared to the LKB NTCP model (R_s_ = 0.143, p < .001, AUC = 0.595). The obtained results confirm the importance of including, besides the dose, clinical factors such as the use of anticoagulant and/or antihypertensive drugs and the appearance of acute toxicity to obtain a robust prediction of the late toxicity risk. Models that take into account relationships among different patient-related and dosimetric factors may offer a powerful approach to the optimization of risk ascertainment in order to establish tailored strategies for a patient adapted RT.

Using our cohort of patients, we estimated the best fit values for the parameters of the traditional LKB model by the Maximum Likelihood (ML). The LKB fitted parameter values estimated from our cohort resulted to be *D*_*50*_ = 87.3 Gy, *m* = 0.37, and *n* = 0.10. We compared this result with that of Gulliford et al. for similar endpoints [7]: the *m* parameter resulted comparable, while we found different values for *D*_*50*_ and *n* values. Indeed, Gulliford et al. report *D*_*50*_ value in the range of 54.1-62.6 Gy, *m* value in the range of 0.34-0.6 and *n* value in the range 0.36-0.40.

Very recently, Ospina et al. [5] found lower *n* values (0.003 to 0.06) for all types of late rectal toxicity (grade ≥ 2) showing the serial behavior of rectum. The slight differences in NTCP parameters may depend on several factors, such as the DVH shape, the conformity of the technique, the dose-volume constraints, the dose calculation algorithm and the rectal volume definition [5]. A volume-exponent of *n* = 0.10 reflects a remarkable influence of the high-dose region, which is mostly determined by the shape of the rectum near the prostate. Several studies explore the effects of the setup error and organ motion on the deviations in the dose delivery and the predicted complications uncertainty [27-30]. Of note, in this study no motion-inclusive dose distribution has been used to take into account the variation between the planned dose distribution to the rectum and the treated dose distribution. A recent study reports the differences in associations for tenesmus and rectal bleeding using the planned over the motion-inclusive dose distributions by a simulations of random and/or systematic motion to the planned dose distributions [27]. Except for the uncertainties arising from setup errors, volume organ variability and contouring method, from our results the rectum seems to act as a more “serial-like” organ. The values of *D*_*50*_ = 87.3 Gy (CI 95%, 75.9-102.2 Gy) are similar to literature values for G1-2 rectal bleeding [2,13,31,32] and for stool frequency and fecal incontinence of more severity grade [9].

We expected that the dose response for mild grade complications be shifted to lower dose while the dose response for severity grade of toxicity be shifted to higher doses. However, this expectation was not confirmed by the results. Similarly, Michalski et al. [25] found that the LKB parameters relative to grade ≥ 3 late rectal bleeding were broadly similar to those relative to grade ≥ 2 late rectal bleeding. They attributed this observation to the deviation of daily rectal position from the position of the simulation, which would lead to higher cumulative rectal DVHs than planned.

In this framework, AAPM Task Group 166 suggested great caution to use phenomenological models as representative of radiobiological model of the rectum due to the complex and different clinical situations from which they were derived.

An increased dependence on the highest dose for moderate rectal toxicity of grade 1-2 is in accordance with a smaller *n* value [33]. This effect is evidenced in Figure 2.b. Our results suggest a serially constructed organ when moderate late toxicity endpoints are considered. As shown in Figure 3, the correlation coefficient for the LKB NTCP values decrease as the fractional volume increases, implying that the high-dose parts of the patient’s histograms corresponds to a better representation of the patients’ actual risk. A similar result was already reported by Dale et al. [34] who observed an increased correlation coefficient as *n* decreases.

## Conclusions

In this study, we demonstrated that the multivariate NTCP model deriving from a previous work outperformed the LKB model derived from the same set of patients. In future perspective both models have to be tested on dose distributions including the changes in rectum shape based on multiple CT scans. A dose-volume effect analysis, with and without including rectal motion, will eventually disclose whether the rectal motion model improves the prediction of GI toxicity by the logistic model.

## Abbreviations

GI: Gastrointestinal
RT: Radiation therapy
NTCP: Normal tissue complication probability
LKB: Lyman-Kutcher-Burman
AUC: Area under the curve
G1-2: Grade 1-2
PSA: Prostate specific antigen
DVH: Dose volume histogram
CRT: Conformal radiation technique
ART: Dynamic arc radiation technique
CT: Computed tomography
CTV: Clinical target volume
PTV: Planning target volume
RTOG: Radiation Therapy Oncology Group
EORTC: European Organization for Research and Treatment of Cancer
R_s_: Spearman’s rank correlation
gEUD: generalized equivalent uniform dose
ML: Maximum Likelihood
LLH: Logarithm of the likelihood
AH/AC: Antihypertensive and/or anticoagulant
ROC: Receiver operator characteristic
SD: Standard deviation
